# CDKN2B-AS1 participates in high glucose-induced apoptosis and fibrosis via NOTCH2 through functioning as a miR-98-5p decoy in human podocytes and renal tubular cells

**DOI:** 10.1186/s13098-021-00725-5

**Published:** 2021-10-14

**Authors:** Min Xiao, Shoujun Bai, Jing Chen, Yaxi Li, Shu Zhang, Zhao Hu

**Affiliations:** 1 Department of Nephrology, Qilu Hospital, Cheeloo College of Medicine, Shandong University, 107 Wenhua West Road, Jinan, 250012 Shandong China; 2 Department of Nephrology, Qingpu Branch of Zhongshan Hospital Affiliated To Fudan University, Shanghai, 201700 China; 3 School of Basic Medical Sciences, Ningxia Medical University, Yinchuan, 750004 China; 4 Department of Biomedical Engineering, University of Houston, Houston, TX 77004 USA

**Keywords:** DN, CDKN2B-AS1, miR-98-5p, NOTCH2

## Abstract

**Background:**

Diabetic nephropathy (DN) is the most common causes of end-stage renal disease. Long non-coding RNA cyclin-dependent kinase inhibitor 2B antisense RNA 1 (CDKN2B-AS1) is connected with the development of DN, but the role of CDKN2B-AS1 in DN has not been entirely elucidated.

**Methods:**

Quantitative real-time polymerase chain reaction (qRT-PCR) was carried out to measure CDKN2B-AS1 and miR-98-5p levels. Cell viability, proliferation, and apoptosis were analyzed with 3-(4, 5-dimethylthiazol-2-yl)-2, 5-diphenyltetrazolium bromide (MTT) or flow cytometry assays. Protein levels were measured by western blotting. The relationship between CDKN2B-AS1 or notch homolog 2 (NOTCH2) and miR-98-5p was verified via dual-luciferase reporter assay.

**Results:**

CDKN2B-AS1 and NOTCH2 were upregulated in the serum of DN patients and high glucose-disposed human podocytes (HPCs) and human renal tubular cells (HK-2), whereas miR-98-5p was downregulated. High glucose repressed viability and accelerated apoptosis of HPCs and HK-2 cells. CDKN2B-AS1 knockdown impaired high glucose-induced apoptosis and fibrosis of HPCs and HK-2 cells. Mechanistically, CDKN2B-AS1 sponged miR-98-5p to regulate NOTCH2 expression. Also, CDKN2B-AS1 inhibition-mediated effects on apoptosis and fibrosis of high glucose-disposed HPCs and HK-2 cells were weakened by miR-98-5p inhibitor. Also, NOTCH2 knockdown partly reversed miR-98-5p inhibitor-mediated impacts on apoptosis and fibrosis of high glucose-disposed HPCs and HK-2 cells.

**Conclusion:**

High glucose-induced CDKN2B-AS1 promoted apoptosis and fibrosis via the TGF-β1 signaling mediated by the miR-98-5p/NOTCH2 axis in HPCs and HK-2 cells.

**Supplementary Information:**

The online version contains supplementary material available at 10.1186/s13098-021-00725-5.

## Highlights

1. CDKN2B-AS1 was upregulated in DN and high glucose-induced HPCs and HK-2 cells.
2. Inhibition of CDKN2B-AS1 alleviated high glucose-induced apoptosis and fibrosis of HPCs and HK-2 cells.
3. CDKN2B-AS1 regulated NOTCH2 expression via acting as a miR-98-5p sponge.

**Supplementary Information:**

The online version contains supplementary material available at 10.1186/s13098-021-00725-5.

## Introduction

Diabetic nephropathy (DN), a progressive kidney disease caused by diabetes, is characterized by persistent albuminuria and a gradual decline in estimated glomerular filtration rate [1, 2]. It is reported that about 30–40% of patients with diabetes may develop DN, and approximately 50% of DN patients tend to develop end-stage renal disease [3]. Renal fibrosis is the main driving force for the occurrence of DN, and hyperglycemia in diabetic patients may trigger renal fibrosis [4]. Therefore, exploring the pathogenesis of DN is important to improve DN.

Long non-coding RNAs (lncRNAs) are a class of transcripts that do not have protein-encoding capabilities [5]. Studies have shown that lncRNAs are associated with a plethora of cellular functions [6, 7]. Also, lncRNAs play their roles in a series of diseases through interacting with microRNAs [8–10]. For instance, lncRNA GAS5 decreased pyroptosis and oxidative stress via sponging miR-452-5p in high glucose-induced renal tubular cells [11]. Cyclin-dependent kinase inhibitor 2B antisense RNA 1 (CDKN2B-AS1) is connected with the development of diabetes [12], coronary heart disease [13], atherosclerosis [14], and cancers [15]. Furthermore, CDKN2B-AS1 modulated extracellular matrix accumulation and proliferation of mesangial cells [16]. MiRNA-98-5p (miR-98-5p) plays vital roles in the advancement of numerous diseases. In diabetes, miR-98-5p accelerated cell apoptosis and impeded cell proliferation in keratinocytes through targeting PPP1R15B [17]. MiR-98-5p could improve OGD/R-induced neuronal injury via downregulating Bach1 [18]. Moreover, miR-98-5p mediated renal fibrosis and epithelial-to-mesenchymal in DN [19]. Nevertheless, it is unclear whether CDKN2B-AS1 mediates the development of DN via miR-98-5p.

It has been confirmed that the NOTCH pathway mediates renal fibrosis [20, 21]. Notch homolog 2 (NOTCH2) is one of the important receptors in the NOTCH pathway [22]. NOTCH2 was reported to be connected with high glucose-stimulated cardiac fibrosis and epithelial-to-mesenchymal in HAVECs [23]. Also, JNK is an upstream effector of NOTCH2 in TGF-β1-mediated renal fibrosis [24]. Furthermore, HG triggered EMT through the Notch2 pathway in NRK-52E cells [25]. However, the regulatory mechanisms associated with NOTCH2 in DN development have not been fully elucidated.

Herein, we reported an accelerative influence of CDKN2B-AS1 on the pathogenesis of DN. Also, we found that CDKN2B-AS1 induced apoptosis and fibrosis through upregulating NOTCH2 via sponging miR-98-5p under high glucose treatment. Therefore, the research provided a novel mechanism to comprehend the pathogenesis of DN.

## Materials and methods

### Subjects

The research was authorized and supervised by the ethics committee of Qilu Hospital, Cheeloo College of Medicine, Shandong University. 30 patients with DN and 30 healthy controls were recruited from Qilu Hospital, Cheeloo College of Medicine, Shandong University. T2D patients with a urine albumin/creatinine ratio > 30 mg/g or estimated glomerular filtration rate (eGFR) < 60 mL/min/1.73 m^2^ were defined to have DN. The participating patients were free of cardiovascular disease, chronic liver disease, or cerebrovascular disease. Participants in this study signed informed consent.

### Cell culture, treatment, and transfection

Human podocytes (HPCs) and human renal tubular cells (HK-2) (Bena Culture Collection, Suzhou, China) were cultured in Dulbecco’s Modified Eagle’s Medium (Sigma, St Louis, MO, USA) with 10% fetal bovine serum (FBS, Life Technologies, Carlsbad, CA, USA), streptomycin (100 μg/mL, Life Technologies), and penicillin (100 U/mL, Life Technologies) at 37 °C under an atmosphere containing 5% CO_2_. For high glucose treatment, HPCs and HK-2 cells were treated with 30 mM glucose for 24 h. 5 mM glucose acted as a normal glucose, whereas 5 mM glucose plus 25 mM mannitol acted as an osmotic control.

Small interference RNA targeting CDKN2B-AS1 (si-CDKN2B-AS1) and NOTCH2 (si-NOTCH2), as well as their matching controls (si-NC), were obtained from GenePharma (Shanghai, China). The sequences of CDKN2B-AS1 and NOTCH2 were cloned into the plenti-GIII-CMV-2A-Puro-GFP vector (vector) (ABM, Canada) or pcDNA3.1 vector (pc-NC) (Invitrogen, Carlsbad, CA, USA) to obtain plenti-GIII-CMV-2A-Puro-GFP-CDKN2B (CDKN2B-AS1) and pcDNA3.1-NOTCH2 (pc-NOTCH2) vectors, respectively. MiR-98-5p mimic and inhibitor, as well as their corresponding controls (miRNA NC and inhibitor NC), were also bought from GenePharma. When cell confluence reached 80%, HPCs and HK-2 cells were transfected with the designated plasmids or oligonucleotides using Lipofectamine 3000 reagent (Life Technologies).

### Quantitative real-time polymerase chain reaction (qRT-PCR)

Total RNA was extracted through TRIzol reagent (Life Technologies). Total RNA was reverse-transcribed by PrimeScript RT reagent Kit (Takara, Dalian, China) or miRNA First-Strand Synthesis Kit (Takara). QPCR was conducted through the SYBR Premix Ex Taq (Takara) with specific primers (Table1, β-actin and U6 were utilized as house-keeping genes). Expression levels of CDKN2B-AS1 and miR-98-5p were figured with the 2^−ΔΔCt^ method.

**Table 1:** Primer sequences for qRT-PCR

Genes	Primer sequences (5ʹ-3ʹ)
CDKN2B-AS1	Forward (F): 5ʹ-CTATCCGCCAATCAGGAGGC-3ʹ
	Reverse (R): 5ʹ-AAAAGGGACACTAGTCCGGC-3ʹ
miR-98-5p	F: 5ʹ-CGCGCGTGAGGTAGTAAGTTGT-3ʹ
	R: 5ʹ-AGTGCAGGGTCCGAGGTATT-3ʹ
β-actin	F: 5ʹ-TGGATCAGCAAGCAGGAGTA-3ʹ
	R: 5ʹ-TCGGCCACATTGTGAACTTT-3ʹ
U6	F: 5ʹ-GCTTCGGCAGCACATATACTAAAAT-3ʹ
	R: 5ʹ-CGCTTCACGAATTTGCGTGTCAT-3ʹ

### 3-(4, 5-dimethylthiazol-2-yl)-2, 5-diphenyltetrazolium bromide (MTT) assay

Cell viability and proliferation were determined with an MTT assay kit (Beyotime, Shanghai, China). After a period of incubation, the MTT solution (20 μL, 5 mg/mL) was added to each well and incubated for 4 h. After moving the medium, the dimethylsulfoxide (150 μL) was used to solubilize the crystals. The optical density at 490 nm was analyzed through a Microplate Reader (Bio-Rad, Richmond, CA, USA).

### Cell apoptosis analysis

After collection, digestion, and centrifugation, the cells were re-suspended in binding buffer (1×). Cell apoptosis was analyzed using the Annexin V-fluorescein isothiocyanate (FITC)/PI apoptosis detection kit (KeyGen, Jiangsu, China). Cell fluorescence was analyzed through a FACScan flow cytometry (Beckman Coulter, Brea, CA, USA).

### Dual-luciferase reporter assay

The sequences of wild type (WT) CDKN2B-AS1, mutant (MUT) CDKN2B-AS1, WT-NOTCH2-3ʹUTR, and MUT-NOTCH2-3ʹUTR were inserted into the pmirGLO luciferase vectors (GeneCreat, Wuhan, China), respectively. HPCs and HK-2 cells were transfected with a luciferase reporter together with miRNA NC or miR-98-5p mimic. The luciferase intensities were assessed via the luciferase reporter assay kit (Promega, Madison, WI, USA).

### Western blot analysis

Total protein was extracted with the RIPA buffer containing a protease inhibitor cocktail (Sigma). 30 μg total protein was isolated via the sodium dodecyl sulfate-polyacrylamide gel electrophoresis and transferred onto polyvinylidene difluoride (PVDF) membranes (Bio-Rad). Subsequently, the PVDF membranes were incubated with primary antibodies, including rabbit anti-NOTCH2 (ab137665, 1:500, Abcam, Cambridge, MA, USA), rabbit anti-TGF-β1 (ab92486, 1:500, Abcam), rabbit anti-Bax (ab32503, 1:1000, Abcam), rabbit anti-Bcl-2 (ab182858, 1:2000, Abcam), rabbit anti-fibronectin (FN) (ab32419, 1:1000, Abcam), rabbit anti-collagen I (Col.l) (ab34710, 1:2000, Abcam) and rabbit anti-β-actin (ab8227, 1:1000, Abcam). GAPDH was used as a loading control. Next, the PVDF membranes were incubated with goat anti-rabbit IgG (ab97051, 1:5000, Abcam). Protein bands were visualized with an ImmunoStar LD (Wako Pure Chemical, Osaka, Japan). Densitometric analysis was carried out using ImageJ software 1.6.0 (NIH, MD, USA).

### Statistical analysis

The data were expressed as mean ± standard deviation, which was derived from 3 replicate experiments. GraphPad Prism 6.0 software was utilized for statistical analysis. Differences were deemed significant if *P* < 0.05. Student’s *t* test was used to analyze the differences between two groups. One-way variance analysis with Turkey’s test was utilized for the comparison of the differences among more groups.

## Results

### CDKN2B-AS1 was upregulated in DN and high glucose-stimulated HPCs and HK-2 cells

**Fig. 1:**
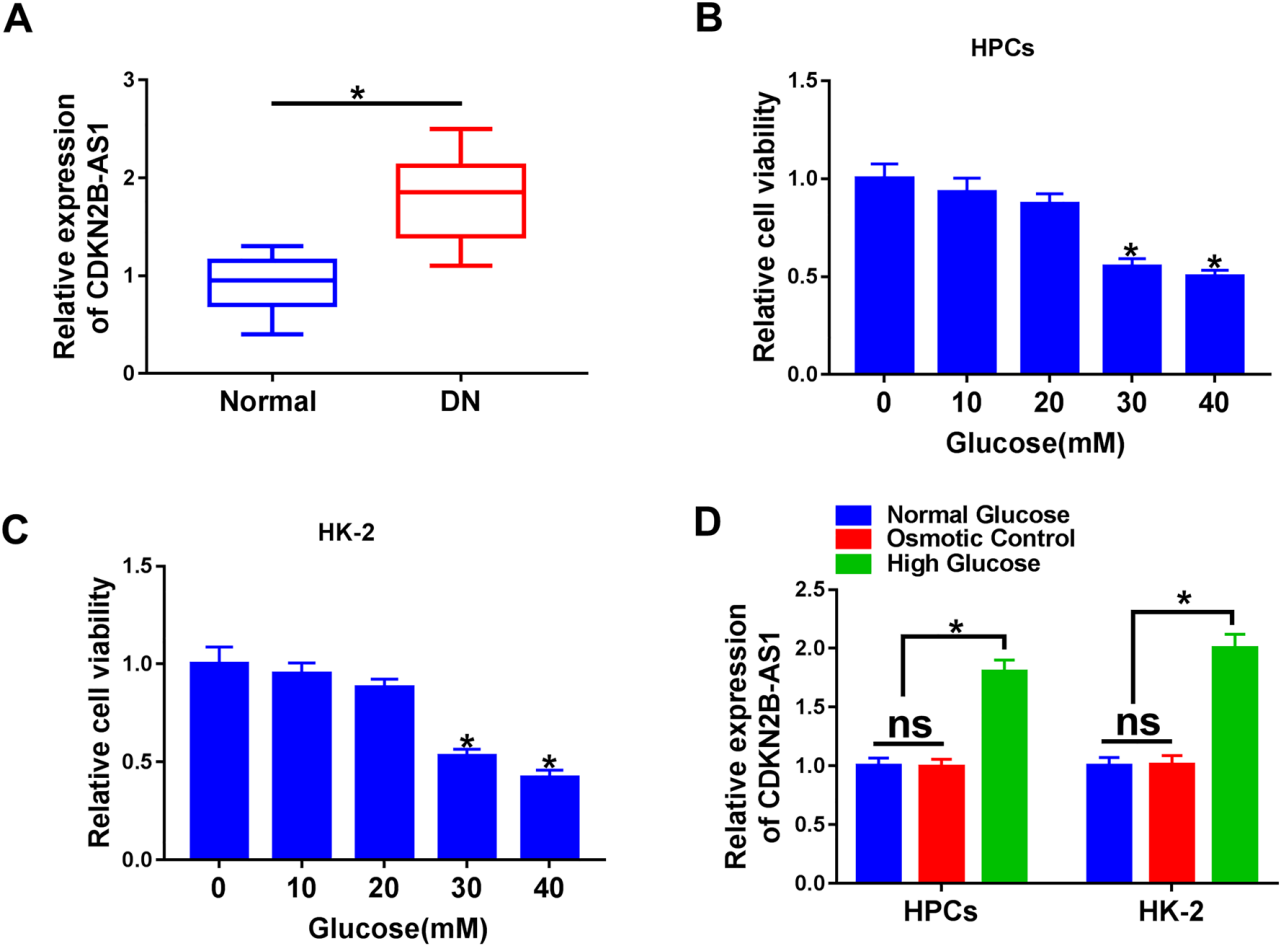
Expression levels of CDKN2B-AS1 in DN and high glucose-disposed HPCs and HK-2 cells. **A** QRT-PCR was performed to analyze the expression levels of CDKN2B-AS1 in the serum of 30 DN patients and 30 normal controls. **B**, **C** MTT assay was conducted for the evaluation of the viability of HPCs and HK-2 cells disposed with different concentrations of glucose (0, 10, 20, 30, and 40 mM). **D** QRT-PCR was executed to assess the expression levels of CDKN2B-AS1 in HPCs and HK-2 cells treated with normal glucose, 5 mM glucose plus 25 mM, and 30 mM glucose. *P < 0.05

Considering the abnormal expression of CDKN2B-AS1 in DN, we detected the expression level of CDKN2B-AS1 in serums from 30 DN patients and 30 normal controls. QRT-PCR manifested that CDKN2B-AS1 expression levels were increased in the serum of DN patients compared to the control group (Fig. 1A). Subsequently, we assessed the viability of HPCs and HK-2 cells treated with different concentrations of glucose. MTT assay presented that high glucose (30 mM and 40 mM) led to a decrease in the viability of HPCs and HK-2 cells (Fig. 1B, C). And the HPCs and HK-2 cells treated with 30 mM glucose were chose for subsequent analysis. We observed that CDKN2B-AS1 expression levels were elevated in high glucose-treated HPCs and HK-2 cells (Fig. 1D). These results indicated that CDKN2B-AS1 might be involved in the development of DN.

### CDKN2B-AS1 regulated apoptosis and fibrosis of HPCs and HK-2 cells under high glucose treatment

**Fig. 2:**
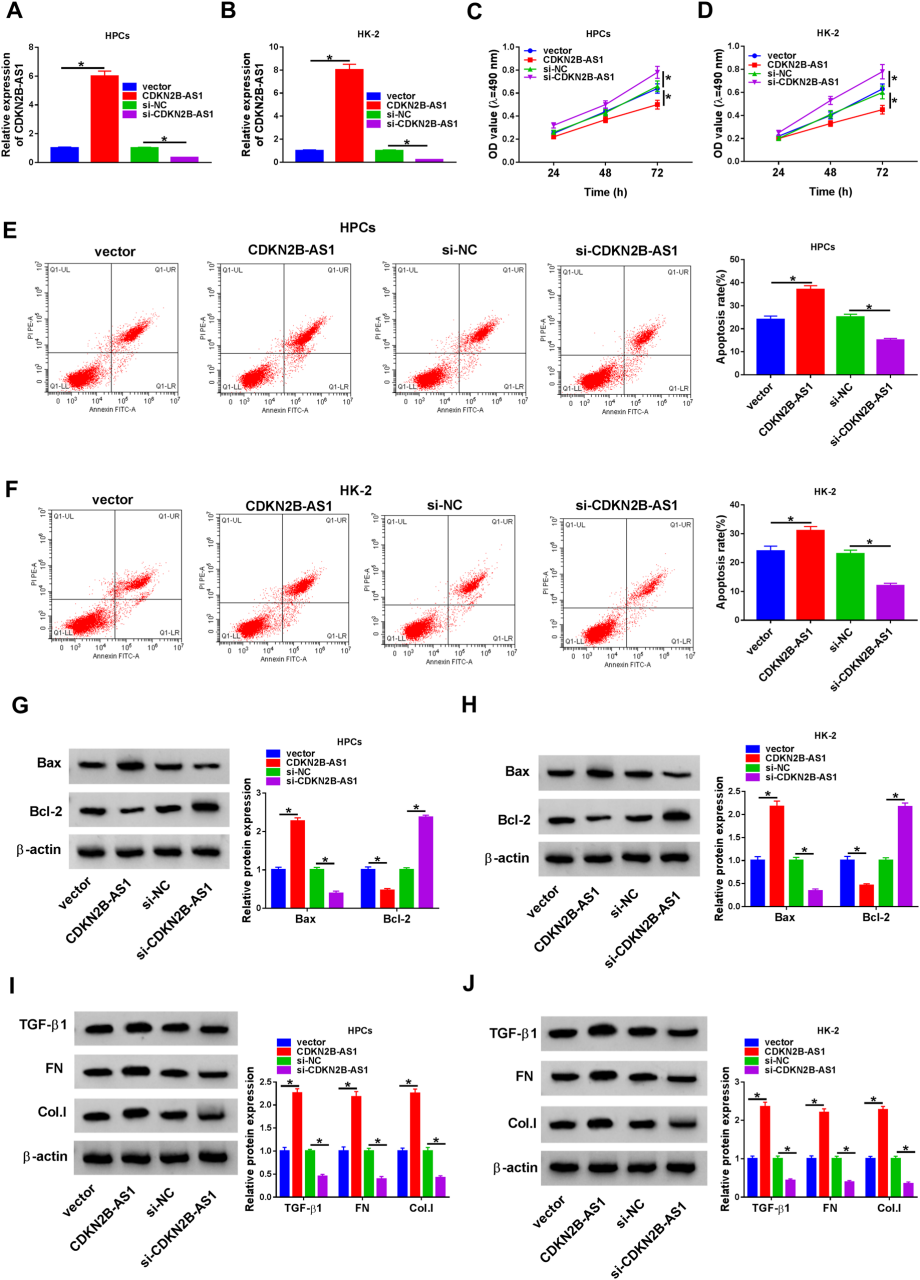
Effects of CDKN2B-AS1 on apoptosis and fibrosis of HPCs and HK-2 cells under high glucose treatment. **A**, **B** The expression of CDKN2B-AS1 in HPCs and HK-2 cells transfected with vector, CDKN2B-AS1, si-NC, or si-CDKN2B-AS1 under high glucose stimulation was analyzed with qRT-PCR. **C**–**F** Effects of CDKN2B-AS1 overexpression and inhibition on the proliferation and apoptosis of high glucose-disposed HPCs and HK-2 cells were determined through MTT or flow cytometry assays. **G**–**J** Effects of CDKN2B-AS1 overexpression and inhibition on protein levels of Bax, Bcl-2, TGF-β1, FN, and Col.I in high glucose-disposed HPCs and HK-2 cells were analyzed by western blot analysis.*P < 0.05

Given that the upregulation of CDKN2B-AS1 in DN and high glucose-disposed HPCs and HK-2 cells, we further investigated the role of CDKN2B-AS1 in DN through loss-of-function experiments. Compared to the control groups, CDKN2B-AS1 was overexpressed in HPCs and HK-2 cells after transfection with CDKN2B-AS1 under high glucose treatment and decreased in HPCs and HK-2 cells after transfection with si-CDKN2B-AS1 under high glucose treatment (Fig. 2A, B). Moreover, CDKN2B-AS1 elevation aggravated proliferation inhibition and apoptosis of high glucose-stimulated HPCs and HK-2 cells, but CDKN2B-AS1 downregulation impaired proliferation inhibition and apoptosis of high glucose-stimulated HPCs and HK-2 cells (Fig. 2C–F). Western blotting displayed that CDKN2B-AS1 elevation resulted in a decrease in Bcl-2 protein levels and an increase in Bax protein levels in high glucose-disposed HPCs and HK-2 cells, while CDKN2B-AS1 silencing played an opposing impact (Fig. 2G, H). In addition, CDKN2B-AS1 overexpression elevation protein levels of TGF-β1, FN, Col.I in high glucose-disposed HPCs and HK-2 cells, but CDKN2B-AS1 silencing decreased protein levels of TGF-β1, FN, Col.I in high glucose-disposed HPCs and HK-2 cells. Collectively, these findings demonstrated that CDKN2B-AS1 regulated apoptosis and fibrosis of HPCs and HK-2 cells under high glucose treatment.

### CDKN2B-AS1 was identified as a sponge for miR-98-5p

**Fig. 3:**
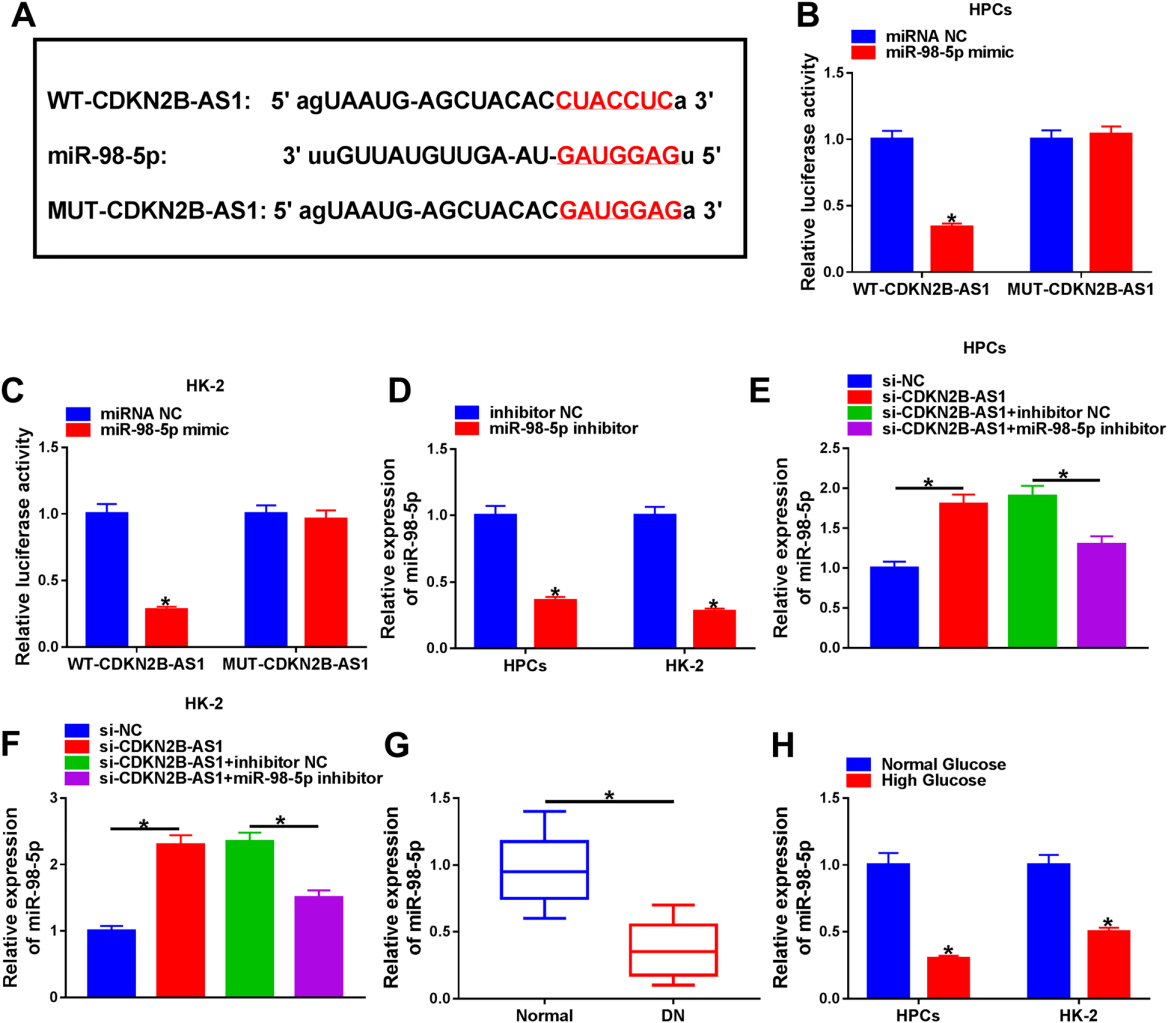
CDKN2B-AS1 acted as a sponge for miR-98-5p. **A** The binding sites between CDKN2B-AS1 and miR-98-5p were predicted by the starBase database. **B**, **C** Dual-luciferase reporter assay was executed for the assessment of the luciferase reporter with WT-CDKN2B-AS1 or MUT-CDKN2B-AS1 in HPCs and HK-2 cells transfected with miR-98-5p mimic or miRNA NC. **D** The expression of miR-98-5p in HPCs and HK-2 cells transfected with miR-98-5p inhibitor or inhibitor NC was detected via qRT-PCR. **E**, **F** Impact of miR-98-5p inhibitor on the expression of miR-98-5p in CDKN2B-AS1-inhibiting HPCs and HK-2 cells was analyzed through qRT-PCR. **G**, **H** The expression of miR-98-5p in serum of DN patients and high glucose-treated HPCs and HK-2 cells was determined via qRT-PCR. *P < 0.05

To explore the underlying molecular mechanism of CDKN2B-AS1 in DN, we predicted the miRNAs that might interact with CDKN2B-AS1 through using the starBase database. MiR-98-5p was discovered to possess a complementary sequence with CDKN2B-AS1 (Fig. 3A). Subsequently, we performed the dual-luciferase reporter assay to verify this prediction. The results exhibited that miR-98-5p mimic repressed the luciferase intensity in HPCs and HK-2 cells with a luciferase reporter containing WT-CDKN2B-AS1, while there was no overt difference in the luciferase reporter containing MUT-CDKN2B-AS1 (Fig. 3B, C). And miR-98-5p expression was decreased in HPCs and HK-2 cells after transfection with miR-98-5p inhibitor (Fig. 3D). Also, CDKN2B-AS1 silencing elevated miR-98-5p expression in HPCs and HK-2 cells, but this suppression was reversed by miR-98-5p downregulation (Fig. 3E, F). Moreover, miR-98-5p was downregulated in the serum of DN patients relative to the normal controls (Fig. 3G). There was a marked reduction in miR-98-5p expression in high glucose-treated HPCs and HK-2 cells (Fig. 3H). These results suggested that CDKN2B-AS1 acted as a miR-98-5p sponge.

### CDKN2B-AS1 regulated apoptosis and fibrosis of high glucose-treated HPCs and HK-2 cells by sponging miR-98-5p

**Fig. 4:**
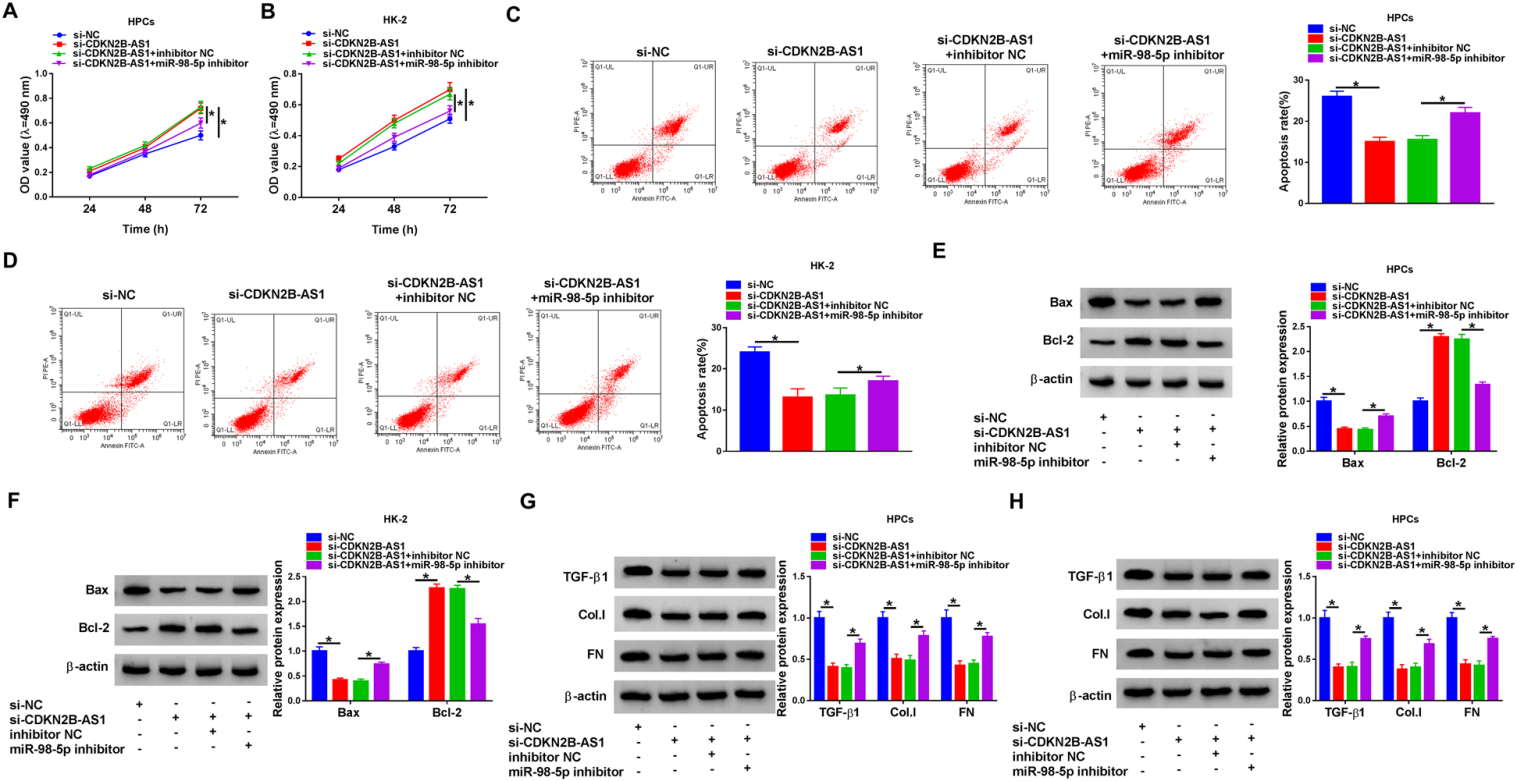
CDKN2B-AS1 regulated apoptosis and fibrosis of high glucose-treated HPCs and HK-2 cells by sponging miR-98-5p. **A**–**H** HPCs and HK-2 cells were transfected with si-NC, si-CDKN2B-AS1, si-CDKN2B-AS1 + inhibitor NC, or si-CDKN2B-AS1 + miR-98-5p inhibitor and then treated with high glucose. **A**–**D** The proliferation and apoptosis of high glucose-treated HPCs and HK-2 cells was detected by MTT and flow cytometry assays. **E**–**H** Protein levels of Bax, Bcl-2, TGF-β1, FN, and Col.I in high glucose-disposed HPCs and HK-2 cells were measured by western blot analysis. *P < 0.05

Subsequently, we further explored whether CDKN2B-AS1 regulated apoptosis and fibrosis of high glucose-treated HPCs and HK-2 cells by sponging miR-98-5p. MiR-98-5p downregulation impaired CDKN2B-AS1 downregulated-mediated effects on proliferation and apoptosis of high glucose-treated HPCs and HK-2 cells (Fig. 4A–D). Also, miR-98-5p inhibitor partially overturned the upregulation of Bcl-2 and the downregulation of Bax, TGF-β1, FN, and Col.I in high glucose-treated HPCs and HK-2 cells caused by CDKN2B-AS1 inhibition (Fig. 4E–H). Together, these results indicated that CDKN2B-AS1 regulated apoptosis and fibrosis of high glucose-treated HPCs and HK-2 cells by acting as a miR-98-5p sponge.

### NOTCH2 was a downstream target of miR-98-5p

**Fig. 5:**
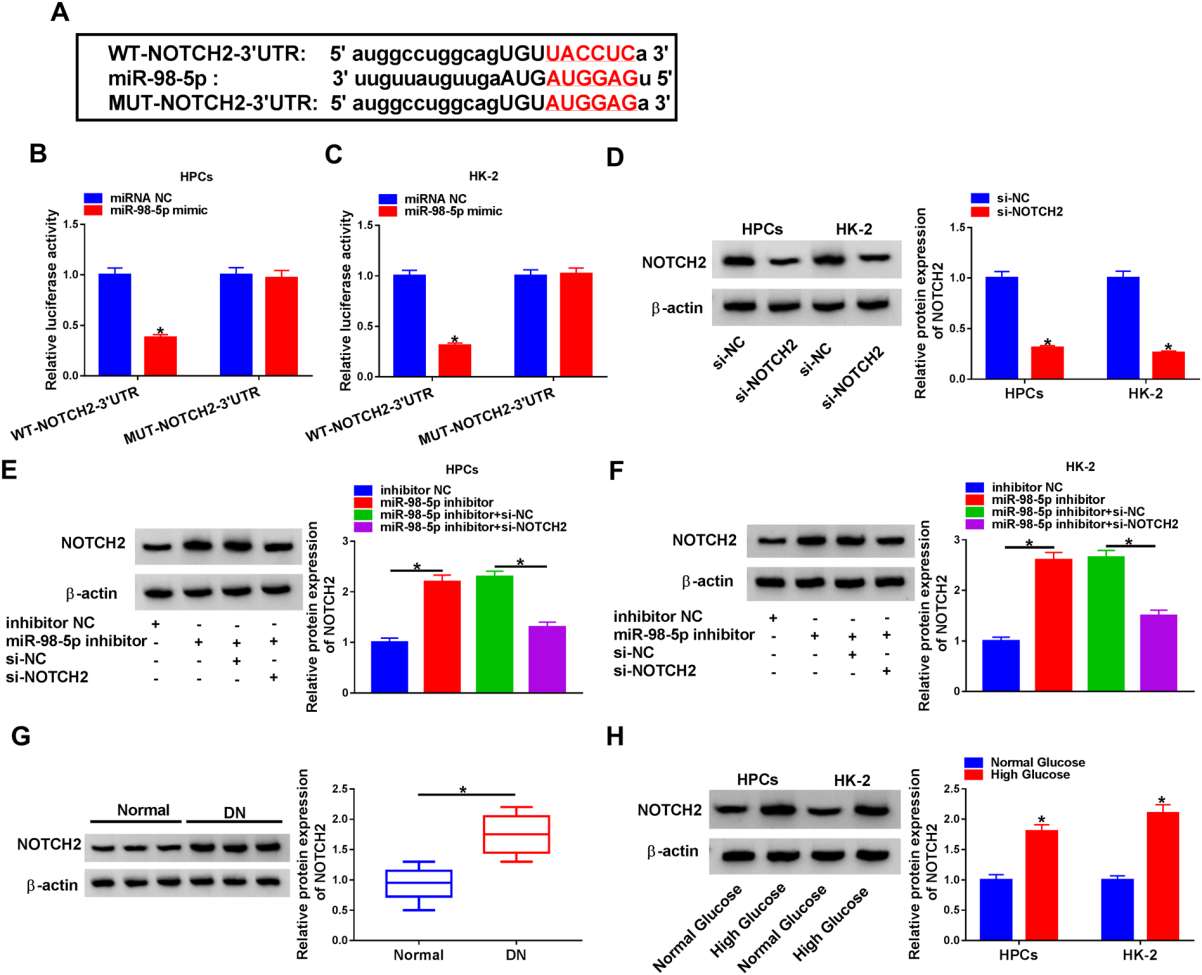
NOTCH2 was a target of miR-98-5p. **A** The binding sites of NOTCH2 in miR-98-5p were predicted by the starBase database. **B**, **C** The luciferase intensity of a luciferase reporter with WT-NOTCH2-3ʹUTR or MUT-NOTCH21-3ʹUTR in HPCs and HK-2 cells transfected with miR-98-5p mimic or miRNA NC was determined through dual-luciferase reporter assay. **D** The protein level of NOTCH2 in HPCs and HK-2 cells transfected with si-NC or si-NOTCH2 was detected with western blot analysis. **E**, **F** Effect of NOTCH2 knockdown on the level of NOTCH2 protein in miR-98-5p-inhibiting HPCs and HK-2 cells was analyzed through western blot analysis. **G**, **H** The protein level of NOTCH2 in the serum of DN patients and high glucose-treated HPCs and HK-2 cells was assessed via western blot analysis

We further predicted the underlying targets of miR-98-5p with the starBase database. And the results presented that NOTCH2 had the complementary base fragment with miR-98-5p (Fig. 5A). The luciferase activity of luciferase reporter with WT-NOTCH2-3ʹUntranslated Regions (UTR) was decreased by miR-98-5p mimic in HPCs and HK-2 cells, while the luciferase intensity of luciferase reporter with MUT-NOTCH2-3ʹUTR did not change (Fig. 5B, C). And the protein levels of NOTCH2 in HPCs and HK-2 cells were markedly restrained after transfection with si-NOTCH2 compared to the control si-NC (Fig. 5D). Moreover, miR-98-5p inhibitor elevated NOTCH2 protein levels in HPCs and HK-2 cells, while this elevation was weakened by NOTCH2 silencing (Fig. 5E, F). Furthermore, NOTCH2 protein levels were also increased in the serum of DN patients and high glucose-treated HPCs and HK-2 cells (Fig. 5G, H). Also, the level of activated NOTCH2 protein was significantly increased under HG conditions (Additional file 1: Figure S1). Collectively, these results indicated that NOTCH2 served as a target of miR-98-5p.

### MiR-98-5p targeted NOTCH2 to regulate apoptosis and fibrosis of high glucose-treated HPCs and HK-2 cells

**Fig. 6:**
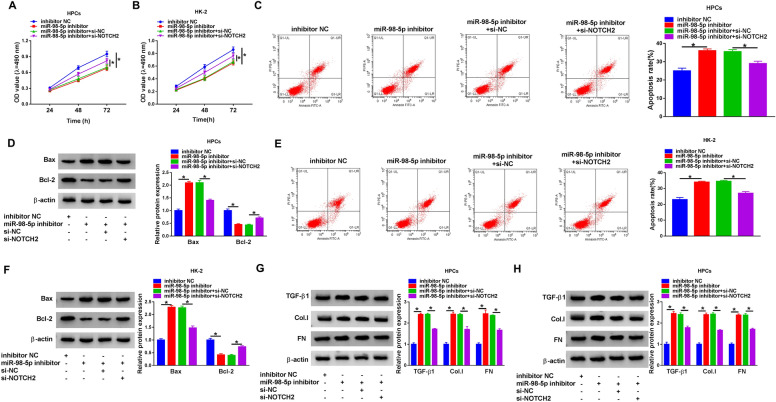
MiR-98-5p regulated apoptosis and fibrosis of high glucose-treated HPCs and HK-2 cells by targeting NOTCH2. **A**–**H** HPCs and HK-2 cells were transfected with inhibitor NC, miR-98-5p inhibitor, miR-98-5p inhibitor + si-NC, or miR-98-5p inhibitor + si-NOTCH2 and then treated with high glucose. **A**–**F** The proliferation, apoptosis, and apoptosis-related proteins of HPCs and HK-2 cells under high glucose treatment were evaluated via MTT assay, flow cytometry assay, and western blotting. **G**, **H** Protein levels of TGF-β1, FN, and Col.I in HPCs and HK-2 cells under high glucose treatment were measured by western blot analysis. *P < 0.05

To determine whether miR-98-5p regulated apoptosis and fibrosis of high glucose-treated HPCs and HK-2 cells through NOTCH2, HPCs and HK-2 cells were transfected with inhibitor NC, miR-98-5p inhibitor, miR-98-5p inhibitor + si-NC, and miR-98-5p inhibitor + si-NOTCH2. The results exhibited that miR-98-5p inhibitor repressed the proliferation of high glucose-treated HPCs and HK-2 cells, while this influence caused by miR-98-5p inhibitor were offset by NOTCH2 knockdown (Fig. 6A, B). In addition, miR-98-5p inhibitor promoted apoptosis, decreased protein levels of Bcl-2, and elevated protein levels of Bax in high glucose-disposed HPCs and HK-2 cells, but these impacts were overturned after NOTCH2 knockdown (Fig. 6C–F). Also, miR-98-5p inhibitor elevated TGF-β1, FN, and Col.I protein levels in high glucose-disposed HPCs and HK-2 cells, but these increases were restored by NOTCH2 silencing (Fig. 6G, H). Taken together, these findings revealed that miR-98-5p regulated apoptosis and fibrosis of high glucose-treated HPCs and HK-2 cells through targeting NOTCH2.

### CDKN2B-AS1 regulated NOTCH2 expression through sponging miR-98-5p

**Fig. 7:**
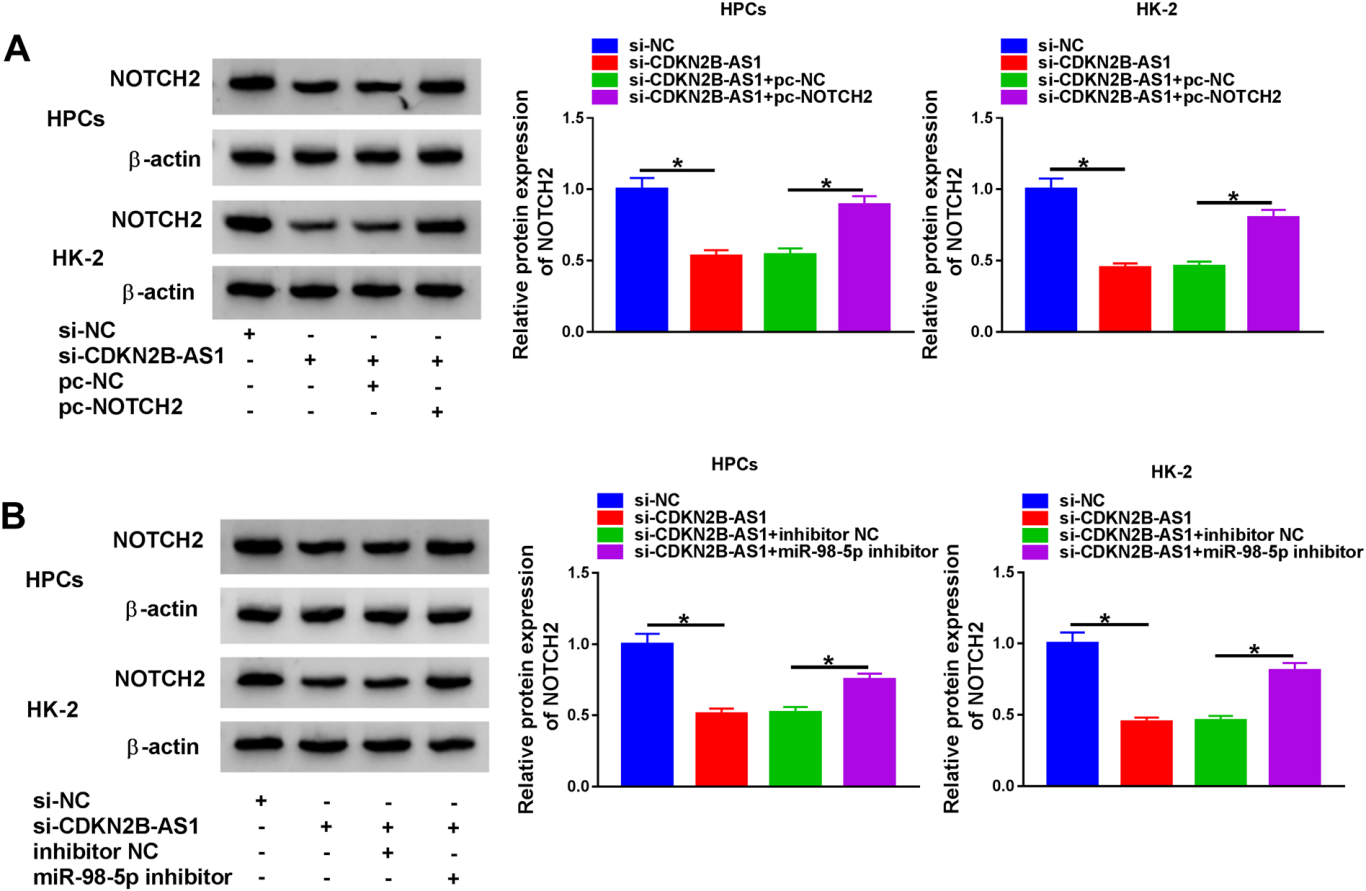
CDKN2B-AS1 modulated NOTCH2 expression through miR-98-5p. **A** Effect of NOTCH2 overexpression on the level of NOTCH2 protein in CDKN2B-AS1-inhibiting HPCs and HK-2 cells under high glucose stimulation was assessed through western blot analysis. **B** Influence of miR-98-5p inhibitor on the level of NOTCH2 protein in CDKN2B-AS1-inhibiting HPCs and HK-2 cells under high glucose treatment was detected through western blot analysis. *P < 0.05

Based on the above findings, we investigated whether CDKN2B-AS1 sponged miR-98-5p to regulate NOTCH2 expression. The results manifested that CDKN2B-AS1 silencing reduced NOTCH2 protein levels in HPCs and HK-2 cells under high glucose treatment, while this decrease was overturned by pc-NOTCH2 introduction (Fig. 7A). Furthermore, miR-98-5p inhibitor weakened the suppressive influence of CDKN2B-AS1 knockdown on NOTCH2 protein levels in HPCs and HK-2 cells under high glucose treatment (Fig. 7B). These results indicated that CDKN2B-AS1 modulated NOTCH2 expression via miR-98-5p.

## Discussion

Persistent proteinuria with or without decreased glomerular filtration rate has been used to define DN [26]. In this study, T2D patients with a urine albumin/creatinine ratio > 30 mg/g or estimated glomerular filtration rate (eGFR) < 60 mL/min/1.73 m^2^ were defined to have DN. In addition, the inclusion of DN patients without renal biopsy was a major limitation of the study.

Podocytes, which constitute the glomerular filtration barrier, have limited regeneration and repair capabilities [27]. Renal tubular injury is an important manifestation of DN [28]. Studies have confirmed that the deregulation of lncRNAs is closely related to the progress of DN [8]. Report of Zhang et al. revealed that lncRNA MALAT1 was overexpressed in high glucose-treated HK-2 cells, resulting in accelerating cell epithelial-to-mesenchymal transition and injury [29]. Another report pointed out that lncRNA MALAT1 increased SIRT1 expression via targeting miR-9, thus alleviating podocyte damage via boosting cell viability and repressing cell apoptosis under high glucose treatment [30]. Lv et al. manifested that lncRNA GAS5 silencing mitigated high glucose-induced viability suppression and apoptosis acceleration of HK-2 cells via sponging miR-27a [28]. In the current study, CDKN2B-AS1 was upregulated in the serum of DN patients and high glucose-treated HPCs and HK-2 cells. Moreover, CDKN2B-AS1 silencing elevated cell viability and decreased cell apoptosis in HPCs and HK-2 cells under high glucose treatment. Thomas et al. demonstrated that CDKN2B-AS1 downregulation protected decreased urine albumin levels and urine volume in diabetic mice [31]. A recent research indicated that CDKN2B-AS1 knockdown inhibited extracellular matrix accumulation and proliferation of high glucose-treated HGMC cells through repressing HMGA2 expression by adsorbing miR-424-5p [16].

TGF-β1 is considered to be the main regulator of pro-fibrosis [32]. Increasing evidence has demonstrated that the TGF-β1 signaling exerts a vital role in DN pathogenesis [33–36]. Moreover, TGF-β1 can contribute to glomerular filtration disorder, fibrosis, and sclerosis [37]. Also, Sitagliptin can block the TGF-beta1/Smad pathway, thus ameliorating diabetic nephropathy [38]. Herein, CDKN2B-AS1 silencing decreased protein levels of TGF-β1, FN and Col.I in high glucose-disposed HPCs and HK-2 cells, indicating that CDKN2B-AS1 silencing decreased the fibrosis of HPCs and HK-2 under high glucose treatment. Thus, we concluded that high glucose-induced apoptosis and fibrosis of HPCs and HK-2 were partly dependent on CDKN2B-AS1.

LncRNAs usually exert their roles through acting as a sponge for miRNAs in DN [16, 28]. A previous study revealed that miR-98-5p repressed human endothelial cell growth through targeting cyclinD2 [39]. Another research reported that miR-98-5p mitigated renal fibrosis and epithelial-to-mesenchymal via modulating HMGA2 expression in DN [19]. Herein, miR-98-5p was downregulated in the serum of DN patients and high glucose-treated HPCs and HK-2 cells. CDKN2B-AS1 was validated as a sponge for miR-98-5p, and the impacts of CDKN2B-AS1 inhibition on proliferation, apoptosis, and fibrosis of high glucose-treated HPCs and HK-2 cells were overturned by miR-98-5p inhibitor. Thus, we concluded that CDKN2B-AS1 played its influence on high glucose-treated HPCs and HK-2 cells via sponging miR-98-5p.

Additionally, NOTCH2 was identified as a miR-98-5p target in the research. Also, NOTCH2 silencing abolished miR-98-5p inhibitor-mediated impacts on proliferation, apoptosis, and fibrosis of high glucose-treated HPCs and HK-2 cells. It was reported that miR-18a-5p targeted NOTCH2 in high glucose-induced HAVECs, thus impeding cardiac fibrosis and epithelial-to-mesenchymal [23]. Furthermore, De-Glycyrrhizinated Licorice Extract blocked the NOTCH2 pathway in high glucose-treated NRK-52E cells, thereby attenuating the epithelial-to-mesenchymal of NRK-52E cells [25]. Importantly, CDKN2B-AS1 regulated NOTCH2 expression via sponging miR-98-5p in the study. Therefore, we inferred that CDKN2B-AS1 silencing could relieve high glucose-induced apoptosis and fibrosis by regulating the miR-98-5p/NOTCH2 axis.

In conclusion, high glucose-mediated CDKN2B-AS1 elevated NOTCH2 expression via adsorbing miR-98-5p, leading to facilitating cell apoptosis and fibrosis in HPCs and HK-2 cells. The study offered a novel mechanism by which CDKN2B-AS1 participated in the pathogenesis of DN.

## Supplementary Information

**Additional file 1: Figure S1.** Western blotting was executed to detection the protein levels of activated NOTCH2 in HPCs and HK-2 cells with normal glucose, osmotic treatment, and HG treatment.

## Data Availability

The analyzed data sets generated during the present study are available from the corresponding author on reasonable request.
