# Supplementary figures and images for: CDKN2B-AS1 participates in high glucose-induced apoptosis and fibrosis via NOTCH2 through functioning as a miR-98-5p decoy in human podocytes and renal tubular cells
Source: Diabetol Metab Syndr. 2021 Oct 14;13:107. doi: 10.1186/s13098-021-00725-5 (PMC8518318; doi:10.1186/s13098-021-00725-5)

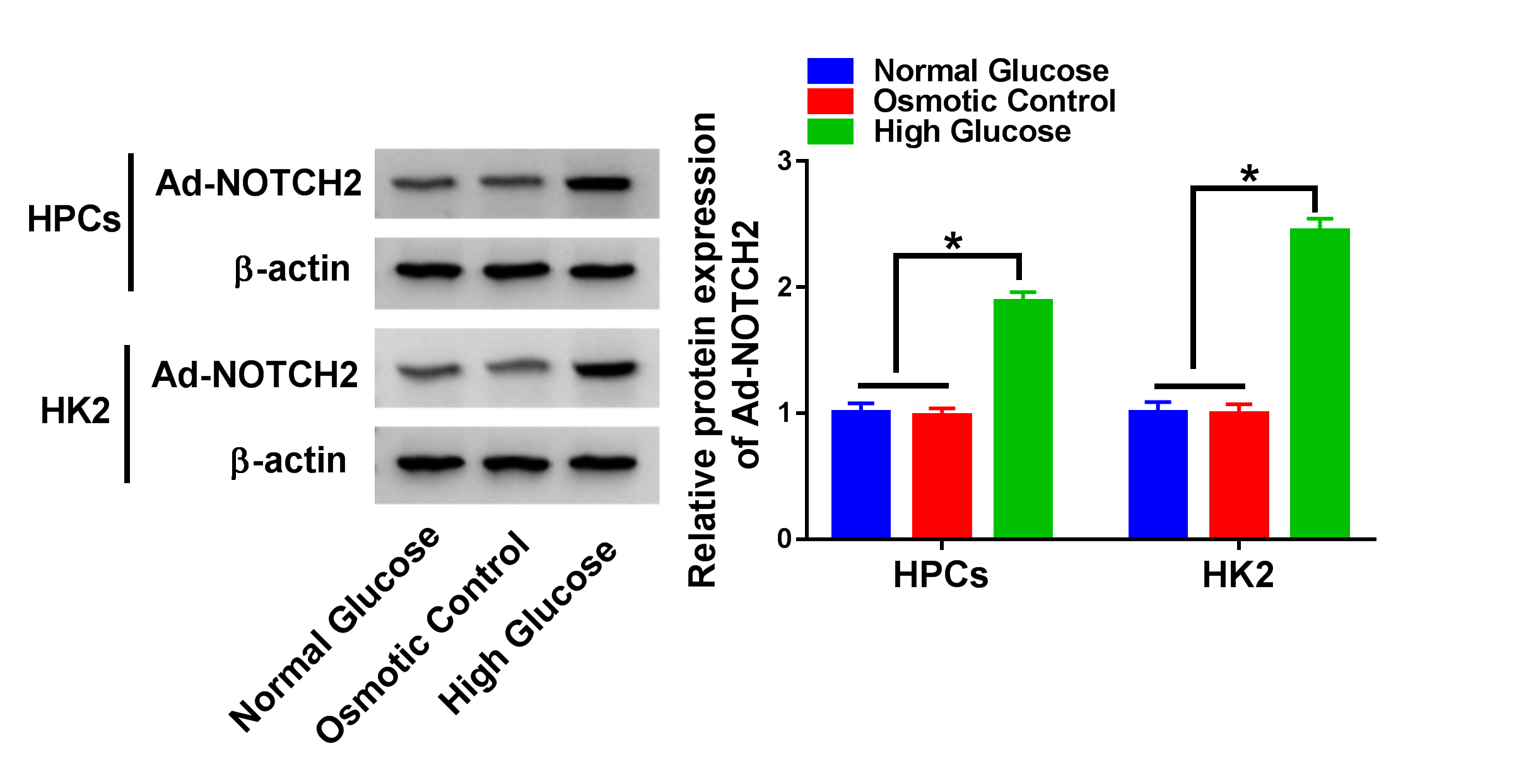

Supplement: Supplementary file 1 — Additional file 1: Figure S1. Western blotting was executed to detection the protein levels of activated NOTCH2 in HPCs and HK-2 cells with normal glucose, osmotic treatment, and HG treatment. [file 13098_2021_725_MOESM1_ESM.tif]
